# Elemental concentrations of ambient particles and cause specific mortality in Santiago, Chile: a time series study

**DOI:** 10.1186/1476-069X-11-82

**Published:** 2012-11-01

**Authors:** Ana Valdés, Antonella Zanobetti, Jaana I Halonen, Luis Cifuentes, Diego Morata, Joel Schwartz

**Affiliations:** 1 Laboratoire de Géosciences Environnement Toulouse (GET), Observatoire Midi-Pyrénées, 14, Avenue Edouard, Belin, Toulouse, 31400, France; 2 Departamento de Geología Aplicada, Servicio Nacional de Geología y Minería de Chile, Avenida Santa María 0104, Providencia, 7520405, Santiago, Chile; 3 Exposure, Epidemiology and Risk Program, Department of Environmental Health, Harvard School of Public Health, Boston, MA, USA; 4 Centro de Medio Ambiente, Escuela de Ingeniería, Pontificia Universidad Católica de Chile, Vicuña Mackenna 4860, Santiago, Chile; 5 Finnish Institute of Occupational Health, Kuopio, Neulaniementie, Finland; 6 Departamento de Geología y Centro de Excelencia en Geotermia de Los Andes (CEGA), Facultad de Ciencias Físicas y Matemáticas, Universidad de Chile, Plaza Ercilla 803, Santiago, 8370450, Chile

**Keywords:** Air pollution, Mortality, PM_2.5_, Elements

## Abstract

**Background:**

The health effects of particulate air pollution are widely recognized and there is some evidence that the magnitude of these effects vary by particle component. We studied the effects of ambient fine particles (aerodynamic diameter < 2.5μm, PM_2.5_) and their components on cause-specific mortality in Santiago, Chile, where particulate pollution is a major public health concern.

**Methods:**

Air pollution was collected in a residential area in the center of Santiago. Daily mortality counts were obtained from the National Institute of Statistic. The associations between PM_2.5_ and cause-specific mortality were studied by time series analysis controlling for time trends, day of the week, temperature and relative humidity. We then included an interaction term between PM_2.5_ and the monthly averages of the mean ratios of individual elements to PM_2.5_ mass.

**Results:**

We found significant effects of PM_2.5_ on all the causes analyzed, with a 1.33% increase (95% CI: 0.87-1.78) in cardiovascular mortality per 10μg/m^3^ increase in the two days average of PM_2.5_. We found that zinc was associated with higher cardiovascular mortality. Particles with high content of chromium, copper and sulfur showed stronger associations with respiratory and COPD mortality, while high zinc and sodium content of PM_2.5_ amplified the association with cerebrovascular disease.

**Conclusions:**

Our findings suggest that PM_2.5_ with high zinc, chromium, copper, sodium, and sulfur content have stronger associations with mortality than PM_2.5_ mass alone in Santiago, Chile. The sources of particles containing these elements need to be determined to better control their emissions.

## Background

Particulate air pollution is a main environmental risk factor for human health, and short-term associations between mortality and particulate pollutants are well established [1-3]. Many studies have suggested that the magnitude of the association between mortality and particles differs by particle size, with fine particles (particles with aerodynamic diameter less than 2.5μm, PM_2.5_) having greater effects than larger particles (diameter between 2.5-10μm, coarse particles) [4,5]. Regional and seasonal differences in the health effects of particles have also been reported [6-8]. Composition of particles also varies by season, suggesting this may play a role in the toxicity of particles. Due to the lack of data on particulate composition, the health effects of specific particulate components have not been widely studied, and most epidemiological studies performed on a population level are from the United States [9-12]. Studies that control for seasonal temperature as a surrogate for ventilation rate have identified sulfur, nickel, and vanadium as particularly toxic [9-11], while studies that ignored confounding by seasonal temperature have reported more mixed results [13,14]. By identifying the elements most toxic to human health, we can move to more efficient regulations for particulate matter. Therefore confirming these associations, particularly in other parts of the world, is important.

In Santiago, Chile, air pollution is a major public health concern because of its dense population and the geography of the area [15]. The city is located between the Andean Cordillera at the East, and Coastal Range at the West. In the Central Valley of Chile, during the majority of the year there is a thermal inversion layer. During autumn and winter, this layer is produced as a result of cooling of the ground. When these phenomena coexist, the conditions became favorable to accumulation of pollution, and the levels of particulate matter regularly exceed the daily standard by U.S. Environmental Protection Agency and World Health Organization (WHO) [16,17]. Previous studies have provided evidence that particulate pollution in Santiago increases the risk of mortality [4,18,19] and morbidity [20,21]. The most recent studies were also able to differentiate the health effects of specific elemental components of particles [18,22]. Unfortunately, as in the U.S., PM_2.5_ mass components in Chile are not measured on a daily basis, hence the data are sparse, and time series analyses have weak statistical power.

We have previously introduced a methodology to take better advantage of sparse data, specifically when speciation data only exist every 3-6 days. PM_2.5_ is monitored more frequently, almost daily. The method was applied to U.S. mortality and morbidity data by Franklin et al. [9] and Zanobetti et al. [11]. In this method, the first stage was fitted on a daily time series analysis by season using daily PM_2.5_ data. In the second stage, we look at how the relative fraction of PM_2.5_, from different elements averaged by season, modifies the PM_2.5_ association. This same approach was subsequently adopted by Bell et al. [10]. In this study we have chosen a similar approach where we let the PM_2.5_ coefficients vary by month, and used the monthly ratios of components to total mass to explain the variations in those coefficients.

We applied this approach to cause-specific mortality during the years 1998-2007. We extracted several different elements of fine particles (aluminium (Al), sodium (Na), silicon (Si), sulfur (S), chloride (Cl), calcium (Ca), chromium (Cr), manganese (Mn), nickel (Ni), potassium (K), iron (Fe), copper (Cu), zinc (Zn), selenium (Se), bromine (Br), lead (Pb)), and studied the associations with mortality for all cardiovascular (CVD), all respiratory, cerebrovascular, and chronic obstructive pulmonary disease (COPD).

## Methods

### Air pollution and meteorological data

The PM_2.5_ mass and elements concentration data were obtained from Parque O’Higgins (P.O), as in prior studies [18,22,23], one of the seven air quality monitoring stations of the Automatic Monitory of Atmospheric Contaminants Network (MACAM NETWORK). This station is located in a residential area in one of the main green areas in the center of Santiago. East of the station is the Principal National Route “Carretera Panamericana" and to the west is the University of Chile Campus. Additionally, in three of the stations temperature, humidity, solar irradiation and wind direction are measured [24].

Particulate matter was collected on 37mm diameter Teflon filters (Pall Flex) [24] by a gravimetric method using a Dichotomous sampler (Sierra Andersen 244, Smyrna, GA). This method allows the collection of particle sizes smaller than 2.5μm (fine fraction), and in the range of 2.5–10μm (coarse fraction) with a bulk flow rate of 16 – 18 l min^-1^. This semiautomatic equipment is programmable for sampling periods of 24 hours, and it allows the simultaneous collection of the two particulate fractions. The samples were collected from 10:00 a.m. to 10:00 a.m. the next day in autumn and winter, and from midnight to midnight in spring and summer. The measurements were performed daily from April to September, every two days in October, November and March, and every three days in December, January and February from 1998 to 2007. The frequency of monitoring is based on the levels of pollution observed during the year and decided by the National Environment Commission. Therefore, daily monitoring in the cold months (April to September) is consistent with higher levels of pollution. Lower pollutant concentrations have been observed in the warm season due to better ventilation conditions and therefore less frequent sampling was performed.

The physical conditioning of the filters was performed in the gravimetric laboratory at the Department of Public Health of the Ministry of Health. Filters were weighted before and after use on an electronic microbalance, Precisa (Swiss) 40SM-200A, allowing 1μg error, and stored in individual plastic boxes in dry chambers. The laboratory atmosphere had a 50% controlled relative humidity and temperature between 20°C and 25°C. The elemental analyses for the PM_2.5_ filters were conducted using X-ray fluorescence at the Desert Research Institute. Six to eight filters per month were analyzed for elements, and approximately 10% of the samples were blank. The limit of detection (LOD) was calculated for each element as three times the standard error of the blanks. Only elements that had at least 95% of all reported values above LOD were included in the statistical analysis, as previously described [18,23].

Based on the results from previous epidemiological studies [18,25] we focused on the species with different sources and toxicological background. We examined the following species: aluminium (Al), sodium (Na), silicon (Si), sulfur (S), chloride (Cl), calcium (Ca), chromium (Cr), manganese (Mn), nickel (Ni), potassium (K), iron (Fe), copper (Cu), zinc (Zn), selenium (Se), bromine (Br), and lead (Pb).

### Health data

Death certificate data in Santiago, with a population around of 6 million inhabitants, was obtained from the National Statistic Institute for the years 1998 to 2007. The causes were classified according to the International Classification of Disease, 9^th^ Revision (ICD-9). We examined daily mortality counts of respiratory diseases (ICD-9: 460-519), cardiovascular diseases (CVD, ICD-9: 390-429), chronic obstructive pulmonary diseases (COPD, ICD-9: 490-496) and cerebrovascular diseases (cerebro, ICD-9: 430-459).

### Statistical methods

We applied a time series analysis using Poisson regression in a generalized additive model to examine the association between daily counts of cause-specific mortality and daily PM_2.5_ mass concentrations. This model adjusts for the over-dispersion of the Poisson-distributed data. The model controlled for seasonality and long term trend with a penalized spline with 5 degrees of freedom (df) for each year; day of the week using indicator variables; the two days average temperature and relative humidity with a penalized spline with 3 df. Because particle species were not measured every day, we computed the mean monthly ratios of the elemental concentrations to the total PM_2.5_ mass for each month therefore eliminating the missing data issue. We first fit a time series analysis of daily PM_2.5_ and daily counts of cause-specific mortality. We then included in the models, one at the time, the interaction terms between the moving average of lag days 0 and 1 of PM_2.5_ and the mean monthly ratio of each individual element to PM_2.5_ mass, the model is:

(1) $$\text{log}\left[E\left(Y_{t}\right)\right]=\beta_{0}+f\left(\text{season}/{\text{time}}_{t}\right)+f\left({\text{temp}}_{t}\right)+f\left({\text{relhum}}_{t}\right)+\beta_{1}\beta_{6}{\text{weekday}}_{6}+\beta_{7}{\text{PM}}_{2.5}+\beta_{8}\text{monthly element concentration}/{\text{PM}}_{2.5}+\beta_{9}{\text{PM}}_{2.5}*\text{monthly element concentration}/{\text{PM}}_{2.5}$$

where, E(Yt) is the expected value of the daily count of mortality Yt, f are the penalized splines of seasonality and long-term trend and weather, β_1_-β_6_ are the coefficients for the weekdays, β_7_ and β_8_ are respectively the main effects of PM_2.5_ and the monthly averages of the element concentrations/PM_2.5_ and β_9_ is the interaction term. This allowed us to see whether the PM coefficient was systematically higher or lower when more (or less) of the PM mass consisted of a particular element.

As those > 65 years of age have been found more susceptible for the effects of air pollution [26], analyses were run separately for this age group. As sensitivity analyses, we ran the models using different degrees of freedom for season, and different lags for the meteorological variables.

The effect estimates are expressed as a percent increase in mortality per 10μg/m^3^ increase in the two-day average PM_2.5_ mass concentration. Because the interaction was determined between two continuous variables we computed the percent increase in cause-specific mortality per 10μg/m^3^ increase in the two-day average PM_2.5_ and for an interquartile (IQR) increase in each monthly average of the element concentrations/PM_2.5_. We used SAS 9.1 [27] for data management, and R 2.7.2 [28] for regression modeling.

## Results

In Santiago, Chile, there were 68,374 deaths from cardiovascular diseases, 24,517 from respiratory diseases, 7,702 from COPD and 22,698 from cerebrovascular diseases over the years 1998 to 2007. Table 1 shows the distribution of the mortality by cause, together with the distribution of the weather variables and PM_2.5_ that had a median 24 hour concentration of 34μg/m^3^. The distributions of the concentration ratios of elements to the total PM_2.5_ mass are presented in Table 2. The largest variations observed in this table (Al, Na, Ca, Cl, Fe, K, S and Si) are associated to elements of natural origin with exception of S, that is probably related to emissions from a large copper smelter in the area [29]. The lower values observed in the rest of the elements probably represent elements of an anthropogenic origin. Table 2 shows also the interquartile range (IQR) of the monthly averages that have been used to compute the percent increase for each element.

**Table 1:** **Distribution of daily mortality by cause**, **weather and PM**_**2**.**5 **_**in Santiago**, **Chile in 1998**-**2007**

	**5%**	**25%**	**50%**	**75%**	**95%**	**N**
Cause of death						
Cardiovascular	11	15	19	23	29	3562
Cerebrovascular	2	4	6	8	11	3562
All respiratory	2	4	6	9	15	3562
COPD	0	1	2	3	5	3562
Environmental variable						
PM_2.5_ μg/m^3^	11	20	34	61	104	2435
PM_2.5_ 2 days average, μg/m^3^	11	18	28	53	96	3204
Temperature °C	8	13	17	21	26	3562
Relative humidity %	34	50	63	75	88	3562

**Table 2:** **Distribution of the element**-**to**-**PM**_**2**.**5 **_**mass proportions and interquartile range** (**IQR**) **of the monthly averages of these ratios**

**Element** (**ng**/**m**^**3**^)	**5%**	**25%**	**50%**	**75%**	**95%**	**N**	**IQR of monthly ratios**
Al	0.55	1.64	3.26	6.68	12.71	816	4.94
Na	0.47	2.36	6.29	14.21	27.71	797	11.42
Br	0.09	0.19	0.31	0.62	2.33	814	0.39
Ca	1.02	2.38	4.00	5.89	9.50	816	3.36
Cl	0.16	0.80	2.20	5.16	15.87	720	4.64
Cr	0.03	0.06	0.10	0.14	0.26	800	0.04
Cu	0.26	0.54	0.75	1.08	1.94	815	0.36
Fe	2.92	6.37	9.07	12.41	18.70	816	5.23
K	3.58	5.97	8.30	12.02	23.43	816	7.23
Mn	0.13	0.29	0.46	0.65	1.04	816	0.21
Ni	0.01	0.02	0.03	0.05	0.09	751	0.02
Pb	0.31	0.64	0.99	2.63	8.24	815	2.74
Se	0.01	0.04	0.08	0.15	0.33	755	0.10
Si	1.91	4.44	8.56	16.42	28.68	816	13.47
S	9.59	22.79	36.72	52.13	81.10	816	22.33
Zn	0.79	1.39	2.05	2.88	4.96	816	0.93

The associations between cause-specific mortality and PM_2.5_ were significant for all of the causes analyzed. The strongest effects were observed for the two-day average and all respiratory mortality with a 1.75% increase (95% CI: 1.01–2.49), and for COPD mortality with 1.94% increase (95% CI: 0.63–3.27) per 10μg/m^3^ increase in PM_2.5_ (Table 3). We also found significant association for cardiovascular and cerebrovascular mortality (Table 3). When PM_2.5_ concentrations were restricted to < 100μg/m^3^ the results didn’t change much, only a slight increase in the effects was observed (data not shown). The effect estimates were slightly greater for those over 65 years of age compared to the whole sample. Again the strongest associations were observed for all respiratory and COPD mortality (Table 3).

**Table 3:** **Percent increase** (**95**% **Confidence interval**) **in cause**-**specific mortality per 10**μ**g**/**m**^**3 **^**increase in the same day and 2**-**day average PM**_**2**.**5**_

**Cause of death**	**Lag**	%	**95**% **CI**	
All				
Cardiovascular	Same day	0.71	0.30	1.13
	2 days average	1.33	0.87	1.78
Cerebrovascular	same day	0.49	−0.22	1.21
	2 days average	1.13	0.36	1.90
All respiratory	same day	0.24	−0.42	0.90
	2 days average	1.75	1.01	2.49
COPD	same day	0.36	−0.83	1.57
	2 days average	1.94	0.63	3.27
Over 65 years of age				
Cardiovascular	same day	0.77	0.32	1.23
	2 days average	1.54	1.05	2.04
Cerebrovascular	same day	0.51	−0.28	1.31
	2 days average	1.29	0.44	2.15
All respiratory	same day	0.41	−0.30	1.13
	2 days average	2.13	1.34	2.93
COPD	same day	0.35	−0.94	1.65
	2 days average	1.95	0.54	3.38

Results for all ages and for age over 65 years.

**Figure 1:**
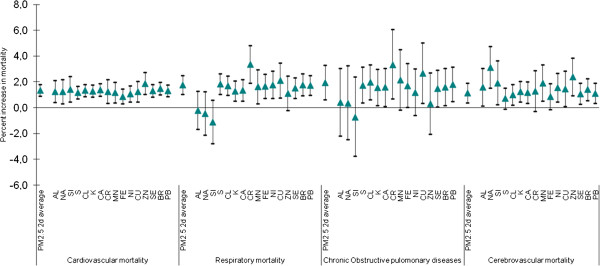
**Percent increase (95% Confidence Interval) in cause-specific mortality per 10μg/m**^**3**^**increases in the 2 days average PM**_**2.5**_**, and for an IQR increase in the elements after including the interaction between PM**_**2.5 **_**and the mean monthly concentration ratios of elements in the total PM**_**2.5 **_**mass.**

Figure 1 shows for each cause of death the results for the two-day average of PM_2.5_ together with the effects of the elements. When we included the interaction term for PM_2.5_ and the elements, we found that a 10μg/m^3^ increase in the two-day average PM_2.5_ and an IQR increase in monthly average of zinc concentration/PM_2.5_ increased the most the effect of PM_2.5_ on cardiovascular (1.87%; 95% CI: 1.04-2.71) and cerebrovascular (2.37%; 95% CI: 0.93-3.83) mortality. Increase in sodium was also associated with cerebrovascular mortality (3.11%, 95% CI: 1.51-4.72).

Chromium and sulfur modified the association between PM_2.5_ and death from all respiratory diseases. A 10μg/m^3^ increase in the two-day average of PM_2.5_ and an IQR increase in monthly average of chromium concentration/PM_2.5_ was associated with increases of 3.35% (95% CI: 1.90–4.83) in all respiratory mortality.

Other elements also had significant associations with all of the outcomes (Figure 1 and Additional file 1 Table S1).

Results of the sensitivity analyses had minor effect on the results (data not shown).

## Discussion

In Santiago, Chile, we found that PM_2.5_ was associated with cause-specific mortality. The effect size per 10μg/m^3^ of PM_2.5_ is similar, but higher to that reported by Zanobetti and Schwartz in an analysis of over 100 U.S. cities [30]. This confirms those associations may be applicable elsewhere. In examining the composition of fine particles, we found that zinc (Zn) was specifically associated with cardiovascular and cerebrovascular deaths, while chromium (Cr) had the strongest associations with respiratory mortality.

The associations we found between PM_2.5_ and cause-specific mortality were consistent with previous studies done in Chile [19,20,31] and in the U.S. [30]. In regard to the elemental concentrations of fine particles, we found a clear relation for all respiratory mortality with Cr; while Zn was associated with higher than average toxicity for both cardiovascular and cerebrovascular deaths. Increased cardiovascular mortality has previously been linked to increases in the levels of elemental and organic carbon, nitrates, sulfates, potassium, copper and iron in California [32]. In line with our findings, an earlier Canadian study has also reported associations between total mortality and zinc [33]. Due to the fact that the PM components have been available only for one day in three or six, some studies in the U.S. have used a method similar to the one presented in this study, and showed that PM chemical components modify the PM effect to mortality [9,12] or hospitalization [10,11]. Among these studies, the elements that have most often been found to increase the health risk are nickel, vanadium, aluminum, arsenic, sulfate, bromine, and silicon.

The effects of particulate species on total mortality in Chile have been studied by Cakmak et al. [18]. They also found associations between mortality and organic carbon (RR 1.07; 95% CI: 1.06-1.07), Cu (RR 1.06; 95% CI: 1.05-1.08) and Fe (RR 1.05; 95% CI: 1.04-1.06), but they only had 655 observations over the nine years of study. Cakmak and co-authors [18] found also that soil-related particles included elements Al, Ca, Fe, and Si, and that these particles had weaker but statistically significant mortality effect.

In an earlier study on the effects of particulate components and sources on emergency department visit counts in Chile, particles related to combustion sources included elements such as Cr, Cu, Fe, Mn and Zn, and they were associated with total and respiratory emergency visits [22]. The association between respiratory morbidity and a factor containing Zn is consistent with the current results; however we found no clear associations between mortality and other soil-related particulate elements Al, Ca, or Fe, which may have been related to the large variation in the levels of these elements. The reasons for different associations between different elements and certain causes of death are to be determined. Ambient concentrations and bio-accessibility may affect the toxicity degree, for example Zn, Mn, and Cu present higher respiratory uptakes than Cd and Pb [34].

The identification of the emission sources of these metals is one of the central problems to discuss. In the current study, sulfur (S) was associated with higher respiratory mortality than the total PM_2.5_ mass, and in a previous study, sulfur has been related with the emissions from a smelter [35]. In fact, one of the potential sources in Santiago is the large copper smelter Caletones that emits oxidized sulfur in the Santiago basin and produces sulfur rich fine particles [29]. Zn has been categorized as a combustion related element along with Cr, Cu, Fe and Mn [18]. The harmful particle sources identified by Cakmak et al. [18] are consistent with the ones previously recognized by Artaxo et al. [36]. They found that the principal source of Zn is oil combustion in particular, while Hedberg et al. [35] and Kavouras et al. [37] related Zn as well as Cu with the copper smelters. Usually, many elements appear to be related to more than one source. For example Fe can be provided by two potential sources: copper smelting processes and/or natural lithogenic source, as suggested by Morata et al. [38]. As already said, Zn also shows this behavior as it appears related to copper smelting and oil/coal combustion. Finally, the presence of Na in Santiago is related to convective process from marine source.

The contradictory results are possibly related to local differences in particulate sources. This underlines the importance of determining the health effects of particulate matter at various locations by elemental components. In Chile there is an ongoing work related with the characterization and identification of particulate sources; however, there are not many studies that show the relationship between public health problems and specimen elements and their sources of emission. As a future prospect, the use of isotopes can help tracing the sources in urban air pollution. This would allow distinguishing different sources associated with specific elements such as Mn, Fe, Zn, S and to discriminate between local sources from the regional, like copper smelters.

There are some limitations to this study. One is that the data collection for the particulate matter was not uniform throughout the year, and daily data was available only for the cold months from April to September. Averaging the elemental concentrations over a month reduced the missing data (measurements daily in winter, every three days in the summer), but may have reduced the variation in the element concentrations, which may have attenuated the strength of the observed associations. However, this would affect the used data equally since the elemental concentrations were similarly assessed in for each month. The air pollution data, was also collected at one measurement location, which may cause some exposure to misclassification. Having data from only one measurement station may lead to Berkson error, which reduces the power to reveal significant effects [39].

Finally, we tested several elemental fractions and outcomes, and thus, the possibility of chance findings due to multiple testing should be considered. However, if one in 20 tests at 95% confidence level are expected to be significant due to chance, [40] our 18 significant findings out of 64 tests (16 elements * 4 outcomes) far exceeds this. The relative strength of the association with the elements needs to be taken with caution and more studies are needed to confirm our findings.

## Conclusions

It seems that PM_2.5_ mass alone is not a sufficient metric when evaluating the health effects of PM exposure. Our findings suggest that particles with high zinc, chromium, copper, sodium, and sulfur content may be related to greater health effects than those observed for the conventionally used measure of total PM_2.5_ mass, in Santiago, Chile. The sources of particles formed of these elements need to be determined in order to better control the emissions of these harmful particulates.

## Abbreviations

PM_2.5_: Particulate Matter with aerodynamic diameter < 2.5μm; Al: Aluminium; Na: Sodium; Si: Silicon; S: Sulphur; Cl: Chloride; Ca: Calcium; Cr: Chromium; Mn: Manganese Ni: nickel; K: Potassium; Fe: Iron; Cu: Copper; Zn: Zinc; Se: Selenium; Br: Bromine; Pb: Lead; CVD: Cardiovascular; COPD: Chronic obstructive pulmonary disease; P.O: Parque O’Higgins; MACAM NETWORK: Automatic Monitory of Atmospheric Contaminants Network; LOD: The limit of detection; ICD-9: International Classification of Disease 9^th^ Revision; E(Y_t_): Value of the daily count of mortality; Y_t_, *f*: Penalized splines of seasonality and long-term trend and weather; β_1_-β_6_: Coefficients for the weekdays; β_7_ and β_8_: Main effects of PM_2.5_ and the monthly averages of the element concentrations/PM2.5; β_9_: The interaction term.

## Competing interests

The authors state that there are no previous publications from the same study in printed or electronic form, and that the paper is not being considered in publication elsewhere. Authors declare no competing interests. The study was not funded or sponsored by industry, or written by a professional medical writer.

## Author's contributions

AV participated to the planning of the study, carried out the chemical analyses, and drafted the manuscript, AZ participated to the planning of the study, analyzed the data, and helped drafting the article, JIH participated to interpretation of data and drafting of the manuscript, LC helped with the acquisition of the data and critically reviewed the manuscript, DM participated to the planning of the study, helped with the acquisition of the data and critically reviewed the manuscript, JS participated to the planning of the study and interpretation of data and critically reviewed the manuscript. All authors have approved the final version of the manuscript.

## Supplementary Material

Additional file 1 **Table S1.** Percent increase (95% Confidence Interval) in cause-specific mortality per 10μg/m^3^ increase in the 2 days average PM_2.5_ , and for 10μg/m^3^ increases in 2 days average PM_2.5_ and an IQR increase in the elements after including the interaction between PM_2.5_ and the mean monthly concentration ratios of elements in the total PM_2.5_ mass. (N=3113) Click here for file
