# Supplementary material for: Elemental concentrations of ambient particles and cause specific mortality in Santiago, Chile: a time series study
Source: Environ Health. 2012 Nov 1;11:82. doi: 10.1186/1476-069X-11-82 (PMC3519772; doi:10.1186/1476-069X-11-82)
Supplement: Additional file 1 — Table S1. Percent increase (95% Confidence Interval) in cause-specific mortality per 10μg/m3 increase in the 2 days average PM2.5 , and for 10μg/m3 increases in 2 days average PM2.5 and an IQR increase in the elements after including the interaction between PM2.5 and the mean monthly concentration ratios of elements in the total PM2.5 mass. (N=3113) [file 1476-069X-11-82-S1.doc]

**Table S1:** Percent increase (95% Confidence Interval) in cause-specific mortality per 10 µg/m^3^ increase in the 2 days average PM_2.5_ , and for 10 µg/m^3^ increases in 2 days average PM_2.5_ and an IQR increase in the elements after including the interaction between PM_2.5_ and the mean monthly concentration ratios of elements in the total PM_2.5_ mass. (N=3113)

|  | **Cardiovascular** | | | | **Respiratory** | | | | **COPD** | | | | **Cerebrovascular** | | | |  |
| --- | --- | --- | --- | --- | --- | --- | --- | --- | --- | --- | --- | --- | --- | --- | --- | --- | --- |
|  | **%** | **95% CI** | | **P-value inter-action** | **%** | **95% CI** | | **P-value inter-action** | **%** | **95% CI** | | **P-value inter-action** | **%** | **95% CI** | | **P-value inter-action** | **IQR** |
|  |  |  |  |  |  |  |  |  |  |  |  |  |  |  |  |  |  |
| PM_2.5_  2 days average | 1.33 | 0.87 | 1.78 |  | 1.75 | 1.01 | 2.49 |  | 1.94 | 0.63 | 3.27 |  | 1.13 | 0.36 | 1.90 |  |  |
|  |  |  |  |  |  |  |  |  |  |  |  |  |  |  |  |  |  |
| AL | 1.24 | 0.40 | 2.09 | 0.91 | -0.22 | -1.69 | 1.28 | 0.01 | 0.39 | -2.19 | 3.03 | 0.25 | 1.58 | 0.14 | 3.04 | 0.31 | 0.0049 |
| NA | 1.22 | 0.29 | 2.16 | 0.91 | -0.47 | -2.13 | 1.23 | 0.01 | 0.34 | -2.48 | 3.25 | 0.34 | 3.11 | 1.51 | 4.72 | 0.00 | 0.0114 |
| SI | 1.41 | 0.43 | 2.41 | 0.64 | -1.12 | -2.79 | 0.58 | 0.00 | -0.75 | -3.77 | 2.37 | 0.09 | 1.91 | 0.23 | 3.61 | 0.23 | 0.0135 |
| S | 1.16 | 0.68 | 1.65 | 0.58 | 1.83 | 1.03 | 2.63 | 0.02 | 1.73 | 0.35 | 3.13 | 0.23 | 0.70 | -0.12 | 1.53 | 0.02 | 0.0223 |
| CL | 1.32 | 0.86 | 1.79 | 0.59 | 1.69 | 0.94 | 2.44 | 0.35 | 1.96 | 0.61 | 3.33 | 0.23 | 1.00 | 0.21 | 1.79 | 0.15 | 0.0046 |
| K | 1.28 | 0.81 | 1.75 | 0.94 | 1.27 | 0.50 | 2.05 | 0.00 | 1.55 | 0.17 | 2.96 | 0.36 | 1.22 | 0.42 | 2.02 | 0.04 | 0.0072 |
| CA | 1.37 | 0.88 | 1.87 | 0.09 | 1.34 | 0.51 | 2.17 | 0.19 | 1.57 | 0.10 | 3.05 | 0.72 | 1.16 | 0.33 | 2.00 | 0.37 | 0.0034 |
| CR | 1.24 | 0.32 | 2.16 | 0.88 | 3.35 | 1.90 | 4.83 | 0.01 | 3.33 | 0.67 | 6.06 | 0.19 | 1.27 | -0.31 | 2.87 | 0.81 | 0.0000 |
| MN | 1.17 | 0.36 | 1.98 | 0.70 | 1.61 | 0.30 | 2.93 | 0.98 | 2.12 | -0.19 | 4.49 | 0.71 | 1.90 | 0.50 | 3.31 | 0.19 | 0.0002 |
| FE | 0.87 | 0.28 | 1.46 | 0.03 | 1.63 | 0.70 | 2.57 | 0.87 | 1.70 | 0.03 | 3.40 | 0.91 | 0.84 | -0.17 | 1.86 | 0.52 | 0.0052 |
| NI | 1.06 | 0.43 | 1.69 | 0.28 | 1.76 | 0.72 | 2.82 | 0.74 | 1.18 | -0.62 | 3.00 | 0.33 | 1.54 | 0.44 | 2.65 | 0.28 | 0.0000 |
| CU | 1.24 | 0.44 | 2.05 | 0.94 | 2.09 | 0.75 | 3.45 | 0.27 | 2.64 | 0.32 | 5.02 | 0.29 | 1.44 | 0.07 | 2.82 | 0.53 | 0.0004 |
| ZN | 1.87 | 1.04 | 2.71 | 0.09 | 1.10 | -0.23 | 2.46 | 0.37 | 0.29 | -2.07 | 2.70 | 0.15 | 2.37 | 0.93 | 3.83 | 0.03 | 0.0009 |
| SE | 1.30 | 0.81 | 1.78 | 0.86 | 1.50 | 0.70 | 2.31 | 0.37 | 1.48 | 0.07 | 2.91 | 0.23 | 1.07 | 0.25 | 1.90 | 0.89 | 0.0001 |
| BR | 1.48 | 0.99 | 1.98 | 0.05 | 1.77 | 0.92 | 2.63 | 0.52 | 1.58 | 0.15 | 3.04 | 0.57 | 1.40 | 0.56 | 2.24 | 0.06 | 0.0004 |
| PB | 1.31 | 0.85 | 1.77 | 0.11 | 1.73 | 0.97 | 2.49 | 0.30 | 1.80 | 0.48 | 3.14 | 0.78 | 1.10 | 0.32 | 1.88 | 0.04 | 0.0027 |
